# The Effects of Temperature on the Development and Survival of *Bathycoelia distincta* (Hemiptera: Pentatomidae), a Significant Pest of Macadamia in South Africa

**DOI:** 10.3390/insects15030152

**Published:** 2024-02-23

**Authors:** Mulalo M. Muluvhahothe, Elsje Joubert, Stefan H. Foord

**Affiliations:** 1 SARChI-Chair on Biodiversity Value and Change, Department of Biological Sciences, Faculty of Science, Engineering and Agriculture, University of Venda, Private Bag X5050, Thohoyandou 0950, South Africa; stefan.foord@univen.ac.za; 2 Levubu Center for Excellence, Private Bag X1001, Levubu 0929, South Africa; elsje@centreforexcellence.co.za

**Keywords:** thresholds, diet, degree days, models, macadamia

## Abstract

**Simple Summary:**

*Bathycoelia distincta* poses a significant threat to the South African macadamia industry. Despite the acknowledged influence of temperature on insects, a comprehensive developmental biology investigation for *B. distincta* has not been undertaken to date. This study systematically presents empirical data on the effects of temperature on the developmental duration, survival, and developmental thresholds of *B. distincta*‘s life stages under constant temperatures. The findings of this study reveal that the thermal developmental range of this pest ranges from 13.5 to 38.0 °C, with an optimum temperature of 29.5 °C. Moreover, *B. distincta* requires 783 degree days to develop from an egg to the adult stage. Considering the prevailing global warming trends, the South African Macadamia industry may experience more damage due to a faster development at warmer temperatures.

**Abstract:**

Temperature is the most influential condition affecting insect development and population dynamics. Understanding its impact and other important factors, such as diet, could provide fine-scale predictions of species abundance and distribution in space and time. The two-spotted stink bug, *Bathycoelia distincta* Distant (Hemiptera: Pentatomidae), is a significant pest of macadamia in South Africa for which limited information on developmental biology exists. Here, for the first time, variations in key developmental parameters of the *B. distincta* biology were studied systematically. The developmental duration, survival rate, development rate, lower developmental threshold (T_min_), optimum developmental threshold (T_opt_), upper developmental threshold (T_max_), and thermal constant were quantified for each developmental stage of *B. distincta*. In addition, the effect of diet (macadamia nut and sweetcorn) on the developmental duration and survival rate were quantified. This study was conducted at five constant temperatures (18, 21, 22, 25, and 29 °C) with relative humidity (RH) variations. The developmental duration from egg to adult decreased significantly with increased temperature: 21 °C (±60 days) to 29 °C (±32 days). The survival rate was significantly different for instar 2 between temperatures. Nymphs (instars 2 and 4) developed faster on the sweetcorn diet than on a macadamia diet, but the total developmental time did not differ significantly between the diets. Development from egg to adult required 783 degree days (DD), with a T_min_ of 13.5 °C, T_opt_ of 29.5 °C, and T_max_ of 38 °C. Ongoing global warming will increase the population growth of *B. distincta* through increased development rate, resulting in more damage to macadamia nuts. Understanding the developmental biology and thresholds for the DD model of *B. distincta* is fundamental for predicting its phenology and outbreaks in macadamia orchards.

## 1. Introduction

Insect development and phenology are crucial in pest control strategies and agriculture in general. Understanding the significance of these factors is essential for effective pest management. Insect phenology refers to the timing of various life stages of insects, such as egg-laying, larval development, pupation, and adult emergence in relation to weather [1]. Knowing when these stages occur is vital for pest control because it allows farmers and pest management professionals to anticipate and target vulnerable life stages [2]. For example, applying pesticides or deploying biological control agents at the right time can effectively reduce pest populations. Inappropriate applications can be costly and reduce natural enemy populations without affecting the target pests [2,3]. Thus, by aligning pest control measures with insect development and phenology, it is possible to minimize the use of chemical pesticides. This reduces the cost of pest management and lowers the risks of pesticide resistance and environmental harm.

Climate change can affect insect phenology by altering temperature and weather patterns. Understanding how these changes impact pest life cycles is essential for adapting pest control strategies to new challenges. Climate change is expected to increase pest number generation and overwintering survival and expand pests’ geographical range [4,5,6]. Crops are becoming increasingly vulnerable to insect pest damage [4,7], resulting in growing concerns for crop protection, production, and food security [8]. There is an increasing need to understand the effects of temperature on pests to improve their management [4,9] because temperature governs insect development and survival, ultimately affecting population size and geographical distribution [10,11]. Although temperature interacts with other abiotic and biotic factors, the effects of temperature on insects are mostly quantified in the laboratory [12] because of the difficulties associated with tracking parameters such as survival and reproduction under field conditions [13].

Due to the ectothermic nature of insects, growers use phenology models to time chemical application [2]. Phenology models require information on the developmental parameters of pests, such as the lower developmental threshold (T_min_) and thermal constant for each life stage expressed in degree days (DD) [14]. Developmental parameters can be derived from the literature based on those of a closely related species [14]. However, closely related species can differ in terms of biological parameters, depending on the geographical area. For example, *Halyomorpha halys* (Stål) (Hemiptera: Pentatomidae) in the United States had a T_min_ of 15 °C [15], whereas the closely related *Antestiopsis thunbergii* (Leston) (Hemiptera: Pentatomidae) in Tanzania had a T_min_ of 12 °C [16]. Thus, determining species-specific biological parameters can add value to effectively managing and optimizing pesticide applications [3].

Insects develop at a linear rate at intermediate temperatures but non-linearly at a wide range of temperatures, with slow development at low temperatures, increasing at higher temperatures before decreasing again [17]. In addition to temperature, factors such as humidity and diet also influence the developmental rate of insects [18,19]. Development is, therefore, driven by a combination of multiple factors that influence other important physiological functions, such as reproduction [20], ultimately affecting species fitness, distribution, and abundance [21,22,23]. Insect developmental rates provide invaluable biological information for understanding species’ seasonality and population trends [15].

The two-spotted stink bug, *Bathycoelia distincta* (Distant) (Hemiptera: Pentatomidae), is a major pest of the South African macadamia industry. Despite its pest status, no systematic developmental study has been conducted for this species except for a degree-day model based on the field temperatures in macadamia orchards in South Africa. *B. distincta* is mainly controlled by pesticide application using calendar dates [24]. Comprehensive knowledge of its developmental biology at different temperatures is needed to improve the degree-day model. This study aims to (i) quantify the effects of temperature (18, 21, 22, 25, and 29 °C) and diet (macadamia nuts and sweetcorn at 25 °C) on the developmental duration and survival of *B. distincta*’s life stages, (ii) fit and evaluate temperature-dependent development models for *B. distincta* life stages, and (iii) determine the developmental thresholds (lower developmental threshold: T_min_, optimum developmental threshold: T_opt_, upper developmental threshold: T_max_, and thermal constant) of *B. distincta*’s life stages.

## 2. Materials and Methods

### 2.1. Stock Colony and Rearing

*B. distincta* eggs were collected from a commercial macadamia farm in Levubu (23°4′0.96″ S, 30°4′31.07″ E), Limpopo, South Africa, from October 2020 to February 2021. The eggs were used to establish a colony under controlled conditions in a laboratory at the University of Venda, Limpopo, South Africa. Upon arrival at the laboratory, four to five egg clusters (≈56 eggs) were placed on a paper towel lined on the bottom of a polypropylene plastic container (45 × 25 × 30 cm) covered with a fine net for ventilation. A small piece of cotton wool moistened with distilled water was placed in each container to increase humidity [25] and provide water for instar 1 nymphs [26]. All nymphal stages were reared in smaller transparent polypropylene containers (45 × 25 × 30 cm) and transferred to larger containers (33 cm × 24 cm × 16 cm) when they transitioned to the adult stage for mating and oviposition. As eggs are oviposited with a glue-like material that attaches them to the oviposition surface, a few drops of water were added to egg clusters and removed with a scalpel. Fresh macadamia nuts were initially provided as a food source, but this was later changed to sweetcorn (*Zea mays*) kernels on the cob to maintain the colony throughout the year. Nuts collected from the orchard were rinsed thoroughly before feeding the colony, and sweetcorn was bought on the same day or a day before feeding. Water and food were replenished two to three times per week. Rearing containers were cleaned twice weekly with a mixture of 50% water and 50% ethanol to prevent fungal infection. The colony was supplemented with wild eggs in 2022 from macadamia orchards to reduce the inbreeding effect. The colony was kept in a cold storage at a temperature of 25 ± 0.0 °C and relative humidity (RH) of 71.8 ± 0.1%, verified and recorded at hourly intervals using Thermocron iButtons (Semiconductor Corporation, Dallas/Maxin, TX, USA) and a photoperiod of 16 L:8 D.

### 2.2. Effect of Temperature on the Developmental Duration and Survival Rate

Three to four newly laid egg packets (N ≈ 42) were collected within 24 h from the stock colony. Eggs were placed in transparent polypropylene containers (45 × 25 × 30 cm), and kept at 18, 21, 22, 25, and 29 °C. This was replicated six times in each temperature treatment. The set temperatures were 18, 20, 22.5, 25, 27.5, and 30 °C, but the actual temperatures and RH in the growth chambers varied as follows: 18.3 ± 0.0 °C and 28.8 ± 0.3% RH (18), 20.8 ± 0.3 °C and 91.2 ± 0.1% RH (20), 22.2 ± 0.0 °C and 29.8 ± 0.2% RH (22.5), 25.3 ± 0.0 °C and 64.1 ± 0.1% RH (high RH) (25), 25.4 ± 0.0 °C and 25.3 ± 0.1% RH (low RH) (27.5), and 29.2 ± 0.0 °C and 22.7 ± 0.3% RH (30). Labcon, LTGC model M20, climate chamber was used for 18 (18), 22.5 (22.2), and 27.5 °C (25_low RH_); Memmert Peltier-cooled model IPP260 for 20 °C (20.8); cold storage for 25 °C (25_high RH_); and Labotec model 356 for 30 °C (29.2). The completion time of each life stage was recorded. The total number of alive and dead were recorded daily. The presence of exuvia signaled the completion of each life stage. Cleaning the containers, the replacement of food, and water supply were similar to the rearing methods, except at 29 °C, where food was changed three times a week. Containers with instar 1 were not cleaned until they reached instar 2 to allow for their aggregation behavior.

### 2.3. Statistical Analyses

All analyses were performed in R version 4.2.1 (R core team, version 4.2.1, 2022). Outliers were identified and removed using the Interquartile Range (IQR) method before the analyses. Means, standard deviations, standard errors, and 95% confidence intervals were calculated using the Rmisc package [27]. The mean for the developmental duration from one stage to another was calculated as a weighted mean. Model assumptions were evaluated by testing for the normality of model residuals using Shapiro–Wilk and Levene’s tests for homogeneity of variance. The effect of temperature on developmental duration and survival rate for normally distributed and homoscedastic data was analyzed using one-way ANOVA followed by Tukey HSD multiple post hoc tests, and non-normal or heteroscedastic residuals were analyzed using a Kruskal–Wallis test followed by Duncan’s multiple post hoc test. The effect of diet on developmental duration and survival rate at 25 °C was analyzed using Student’s *t*-test. The latter analyses were determined using the ggsignif [28] and ggstatsplot R packages [29] on the alpha level = 0.05 (95% confidence interval). Temperature-dependent models (Taylor-81, HarcourtYee-82, Kontodimas-04, and Damos-08) were fitted using the DevRate package [30]. The starting value parameters of all models were obtained from the “Hemiptera” and “Lepidoptera” families in the DevRate package. The “nlsDR” DevRate function was used to calculate the T_min_, T_opt_, and T_max_ from the four non-linear models for each life stage and total development.

### 2.4. Temperature-Dependent Models

Four non-linear models: Taylor-81 [31], HarcourtYee-82 [32], Kontodimas-04 [33], and Damos-08 [17], were fitted to the observed developmental rates of *B. distincta*’s life stages. In all the model equations, rT is the mean developmental rate (1/day), and T (°C) is the temperature (Table 1). 

### 2.5. Developmental Thresholds and Thermal Constant

Non-linear models were used to estimate the T_min_, T_opt_, and T_max_ of *B. distincta*’s life stages. T_min_ is the temperature below which there is no development, T_opt_ is the temperature at which development is maximized, and T_max_ is the temperature above which development stops. The thermal constant expressed in degree days (DD) is the total number of thermal units required to complete a life stage [2].

The thermal constant is calculated as follows:

(1) $$\mathrm{DD}=\left(T-T_{\min}\right)\times D$$

where T represents the examined constant temperature, T_min_ is the lower developmental threshold temperature, and D is the duration of the stage in days, calculated for each life stage [34].

## 3. Results

### 3.1. Effect of Temperature on the Developmental Duration and Survival Rate

The mean developmental duration of all *B. distincta* life stages varied significantly in response to temperature (eggs: ANOVA: F_5,28_ = 88.9, *p* < 0.001; instar 1: ANOVA: F_5,26_ = 53.7, *p* < 0.001; instar 2: ANOVA: F_5,29_ = 22.1, *p* < 0.001; instar 3: ANOVA: F_4,23_ = 8.2, *p* < 0.001; instar 4: χ^2^_Kruskal–Wallis_ (18.1) = 4, *p* = 0.001; instar 5: ANOVA: F_4,22_ = 21.6, *p* < 0.001). At 18 °C, individuals only survived up to instar 2 (Table 2). The longest developmental duration was recorded at 18 °C for eggs (15 days) and the shortest at 29 °C for instar 1 (4.1 days). Total developmental duration from egg to adult varied significantly between temperatures (ANOVA: F_4,22_ = 21.4, *p* < 0.001) with 60.5 days at 20 °C, 62.5 days at 22 °C, 38.4 days at 25 °C_high RH_, 41.6 days at 25 °C_low RH_, and 32.0 days at 29 °C (Table 2). The survival rate of all life stages did not vary between temperatures except for instar 2 (ANOVA: F_4,24_ = 2.91, *p* < 0.05), with the lowest survival of 51% at 22 °C and the highest rate of 82% at 25 °C_low RH_ (Table 3).

### 3.2. Effect of Diet on the Developmental Duration and Survival Rate

Diet had a significant impact on the developmental duration of instars 2 (t_Student_ (10) = −3.1, *p* = 0.01) and 4 (t_Student_ (10) = −2.5, *p* = 0.03). Instars 2 and 4 developed faster on the sweetcorn diet (Table 2). No significant differences were found in developmental time observed between the diets for eggs; instars 1, 3, and 5; and total development from egg to adult stage (Table 2). The survival rate did not differ significantly between diets for all the life stages (Table 3).

### 3.3. Temperature-Dependent Models

**Figure 1:**
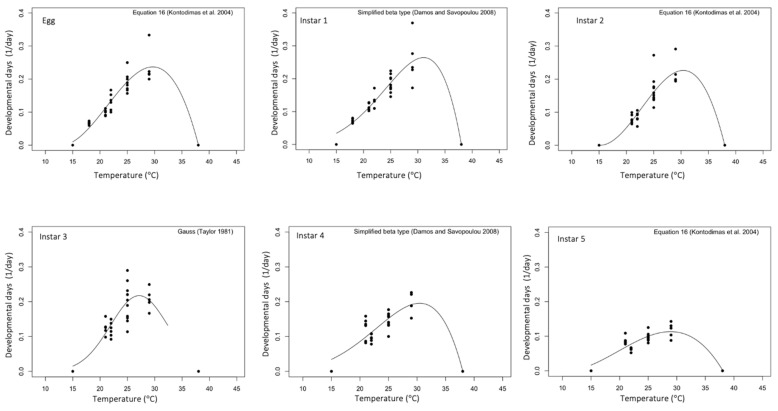
Developmental rate of B. distincta’s life stages at constant temperatures. Each plot describes the best model for each life stage with dots showing the observed rate.

Of all the evaluated models, Kontodimas-04 was the best fit (lowest AIC) for the egg and instars 2 and 5, Damos-08 for instars 1 and 4, and the Taylor-81 model for instar 3 (Figure 1, Table 4).

### 3.4. Developmental Thresholds and Thermal Constant

Non-linear models successfully estimated all developmental parameters (T_min_, T_opt_, and T_max_) for instars 2, 3, 4, and 5 (Table 5). The HarcourtYee-82 model failed to estimate the T_min_ for egg and instar 1, and the Taylor-81 model estimated the T_max_ for egg. Laboratory-based observations suggested that the Kontodimas-04 provided plausible estimates of T_min_ for all life stages and the HarcourtYee-82 model for instars 2 and 3. The best model with the lowest estimate of T_min_ was Damos-08 (instar 4), and the highest was Kontodimas-04 (instar 2). T_opt_ ranged from 27.1 (Taylor-81 model) to 31.8 °C (Damos-08 model) and T_max_ from 38 (HarcourtYee-82, Kontodimas-04, and Damos-08 models) to 46.9 °C (Taylor-81). The Taylor-81 model overestimated the values of T_max_ for all life stages. The Kontodimas-04 was the best model for egg to adult T_min_ (13.5 °C), T_opt_ (29.5 °C), and T_max_ (38.0 °C). The egg’s thermal constant was 62.9 DD: instar 1: 58.9 DD, instar 2: 99.8 DD, instar 3: 134.1 DD, instar 4: 225.7 DD, and instar 5: 201.3 DD. The thermal constant from egg to adult was 783 DD (Table 5).

## 4. Discussion

The developmental duration of all *B. distincta* life stages and total development (egg to adult) significantly decreased at warmer temperatures. The survival rate for all life stages was high but significantly decreased at 22 °C (51%) and peaked at 25 °C_low RH_ (82%) for instar 2. Relative to macadamia nuts, the sweetcorn diet significantly sped up the developmental duration of instars 2 and 4. Temperature-dependent models that best fitted the developmental rate of *B. distincta*’s life stages were Kontodimas-04 (eggs, instars 2 and 5), Damos-08 (instars 1 and 4), and Taylor-81 (instar 3). The Kontodimas-04 model estimated a thermal range of 13.5 °C (T_min_) to 38.0 °C (T_max_) and an optimum developmental rate of 29.5 (T_opt_) for the total development. *B. distincta* required 783 DD to develop from an egg to the adult stage.

This is the first laboratory-based estimate of *B. distincta* development and survival. The developmental durations observed are consistent with those of stink bug species. The incubation period of *B. distincta* was 5.4 days at 25 °C_high RH_, which was very close to the 5.3 days of *Thyanta pallidovirens* (Stål) (Hemiptera: Pentatomidae) [35] at the same temperature. The developmental duration of 7.2 days for instar 2 at 25 °C_low RH_ was also comparable to the 7.4 days of *Nezara viridula* (Linnaeus) (Hemiptera: Pentatomidae) [36]. Notably, the developmental duration of *B. distincta*’s instar 5 was the longest at 22, 25_high RH_, 25_low RH_, and 29 °C, among other life stages. The latter trend was also reported for *Halyomorpha halys* (Stål) (Hemiptera: Pentatomidae) at temperatures ranging from 20 to 33 °C [15] and 20 and 30 °C [37]. The development of sexual organs in instar 5 could explain its prolonged developmental duration. In addition, many insects require substantial amounts of food for the last instar of the immature stage to maximize reproduction [38]. In terms of total development, *B. distincta* took 41.6 days (25 °C_low RH_), which was also observed for *H. Halys* (42 days) [15] at 25 °C. In contrast, at the same temperature, it took *T. pallidovirens* 36 days [35] and *A. thunbergii* 56.53 days to develop from an egg to the adult stage [16].

*B. distincta* completed its full development (egg to adult) at temperatures ranging from 21 to 29 °C. *Chlorochroa uhleri* (Stål) (Hemiptera: Pentatomidae) and *T. pallidovirens* completed their development at temperatures between 20 and 30 °C [35]. These temperature ranges appear to be narrower relative to other stink bug species. For example, *H. halys* and *N. viridula* had a range of 17 to 36 °C and 25 to 36 °C, respectively [20,39]. The wider temperature of *H. halys* is probably related to the larger variation in summer climatic conditions at the higher latitudes of Minnesota, United States [39]. Climate adaptability altered by the geographical regions, genetic variation, and temperatures examined can explain these differences between species.

The survival rate of *B. distincta*’s life stages did not differ significantly between temperatures except for instar 2, contrasting the survival rate of *N. viridula,* which declined with increasing temperature [20]. The eggs of *B. distincta* had the highest survival rate (88–100%) compared to other life stages, suggesting that they may be more temperature-resistant to withstand unfavorable conditions. High survival rates of nymphal stages are associated with the aggregation behavior of newly emerged instar 1 nymphs [40]. Stink bug instar 1 nymphs acquire symbionts by tapping the eggshells using their mouth parts after hatching [41]. Ref. [42] found that the aggregations of *N. viridula* instar 1 nymphs increased humidity, thereby reducing mortality. This could account for the high survival rates of *B. distincta* in this study, as the newly emerged instar 1 nymphs were allowed to aggregate. As a result, it has been suggested that laboratory bioassays start with instar 2 to allow for instar 1 nymph to aggregate [26,42]. A significant drop in survival rate (51%) at 22 °C may possibly be attributed to handling instar 2 with a soft paintbrush when cleaning the rearing containers and changing diets. Considering that *B. distincta* starts dispersing at instar 2, cleaning the containers might have negatively impacted its survival. It is, therefore, necessary to develop alternative methods that require changing the diet without transferring individuals to clean-rearing containers. In contrast, instar 2 was found to be the most thermally plastic stage of *B. distincta* in response to heat and cold hardening and heat acclimation [43], which could be related to its morphology and behavior. The dark pigmentation of this stage could provide more heat absorption when they start searching for food [43,44].

Although diet influences the developmental duration of stink bugs [18], the total developmental duration of *B. distincta* did not differ between the macadamia nut and sweetcorn diet. However, the development of instars 2 and 4 was significantly faster on the sweetcorn diet. Ref. [26] reported the improved development of *H. halys* reared on sweetcorn compared to 15 other diets at 27 °C. It has been shown that stink bug growth can be facilitated by a combination of different diets [26,45]. *B. distincta* is believed to be polyphagous, with adults feeding on a range of different plants, including green tea, sunflower, almond, carrot, *Xylopia* sp., *Bridelia* sp., *Lauracease family*, *Celtis africana*, *Pinus roxburghii*, and *Flindersia* sp. [46]. It takes macadamia nuts about eight months to fully ripen, which could explain why *B. distincta* is prevalent throughout the year in this crop.

Non-linear models suggest that *B. distincta* will develop above 13.5 (T_min_) and below 38 (T_max_), with an optimal developmental rate at 29.5 °C (T_opt_) for the total development from egg to adult. The T_min_ and T_opt_ of the total development of *B. distincta* are consistent with the 13 and 13.2 °C (T_min_) and 29.1 and 28.4 °C (T_opt_) for *T. pallidovirens* and *Chinavia hilaris* (Say) (Hemiptera: Pentatomidae), respectively [35,36]. In contrast, the T_max_ of 30.3 and 33.4 °C of the latter studies are lower than the 38 °C of *B. distincta*, which may be due to California’s cooler climatic conditions and the models used. *B. distincta* can develop at a wide range of temperature regimes in its natural environment based on the temperature developmental thresholds. It has been documented that *B. distincta* occurs throughout the season in macadamia orchards, although the abundance decreases in winter [47,48].

The developmental thresholds estimated for different life stages varied greatly between the models. Similarly, refs. [25,49] reported large variations in developmental thresholds estimated by non-linear models. Despite the differences in the models, non-linear models can overestimate these thresholds irrespective of the temperatures examined [50]. For example, the T_min_ and T_max_ of *B. distincta*’s instar 5 were estimated to be 5.6 °C and 46.8 °C, respectively. Ref. [50] reported an overestimation of the T_max_ and T_min_ for *Bagrada hilaris* (Burmeister) (Hemiptera: Pentatomidae) life stages reared at a temperature range of 20 to 42 °C. Contrary to this study, the T_min_ is mostly determined using linear and non-linear models considering the intermediate temperatures where development is linear. Linear models have been used more often because of their ease of analysis [49,51]. However, some non-linear models can provide stage-specific T_min_, T_opt_, and T_max_ developmental thresholds and more realistic development rate estimations at a wider range of temperatures [31,33]. Given that the temperatures examined (18 to 29 °C) fell within the linear part of development, it is important to note that non-linear models were chosen because there was evidence of a non-linear development of *B. distincta* at a temperature range of 5 to 40 °C (preliminary experiments).

Temperature developmental thresholds strongly influence the potential geographical distribution of a pest [15]. *B. distincta* is currently distributed across the three main macadamia-producing provinces (Limpopo, Mpumalanga, and KwaZulu-Natal) but is dominant in Limpopo, where it was first detected in 1984 [47]. The developmental threshold reported together with the thermal tolerance [43] of *B. distincta* can enable it to expand its range and become more problematic, facilitated by an increased number of hectares of planted macadamia trees annually (9148) and global warming. For example, the ability of *H. halys* to endure extreme temperatures [52,53] could be posited as a plausible factor contributing to its successful documented range of expansion as reported by [54].

The total number of heat units required for *B. distincta* to complete its development (783 DD) was higher than that of other stink bug species like *B. hilaris* (285. 4 DD), *T. pallidovirens* (370.4 DD), *N. viridula* (434.8 DD), *H. halys* (537.6 DD), *C. hilaris* (573 DD), *A. thunbergii* (666.6 DD), and *C. uhleri* (714.3 DD) [15,16,35,36]. A three-year field study on stink bug populations from two macadamia orchards showed that the first nymphal peak of the *B. distincta* required 515 DD while the second peak required 807 DD (Pieter Haasbroek, unpublished data). The latter study used a T_min_ of 10 °C for all life stages to estimate the DD of *B. distincta*, while the current study applied systematically, laboratory-obtained stage-specific T_min_ values, which could explain the differences between the number of DD calculated. Since the DD impacts the seasonal number of generations, it is possible that *B. distincta* completes fewer generations due to its high number of DD.

In conclusion, the macadamia orchard’s ambient mean temperature (five years) was 20 °C with a minimum temperature of 1.0 °C and a maximum temperature of 42 °C for a period of five years [43]. This farm is situated in a dry-winter subtropical highland climate with noticeable dry winters and rainy summers and sometimes experiences extremely hot summers. Thus, *B. distincta*’s population in macadamia orchards will grow rapidly in warmer temperatures, with survival rates not decreasing at temperatures as high as 29 °C. The thermal range of 13.5 (T_min_) to 38 °C (T_max_) with an optimum developmental rate of 29.5 °C (T_opt_) implies that this species can develop at a wide range of temperatures and possibly expand its range as temperatures continue to rise due to global warming.

This study provides insights into determining developmental thresholds that can be useful in improving the existing DD model of *B. distincta*. The life-stage specific T_min_ values can be applied to field-based temperatures to obtain reliable degree days for macadamia farmers. As mentioned above, this pest is solely managed by applying chemicals without simulation models to predict its phenology and population dynamics. Thus, this study can be used as a baseline to understand the phenology of *B. distincta* in macadamia orchards and contribute to its management. To effectively manage the populations of *B. distincta*, in-depth knowledge of its dispersal in and out of the macadamia orchards and its development at a wide range of temperatures can be important. The reported biology of *B. distincta* can be used in decision-making as part of an integrated pest management (IPM) approach to mitigate its damage in the face of global climate change.

## Figures and Tables

**Table 1:** Non-linear models fitted to the developmental rate (1/day) of *B. distincta*’s life stages as a function of temperature.

Models	Equation	Reference
Taylor-81	$\mathrm{rT}\;=R_{m}\times e^{-\frac{1}{2}\times {\left(\frac{T-{\;T}_{m}}{T_{o}}\right)}^{2}}$	Taylor 1981 [31]
HartcourtYee-82	$\mathrm{rT}=a0+a1\times \;T+a2\times T^{2}+a3\times T^{3}$	Harcourt and Yee 1982 [32]
Kontodimas-04	$\mathrm{rT}\;=\mathrm{aa}\;\times {\left(T-{\;T}_{\min}\right)}^{2}\times \left(T_{\max}-T\right)$	Kontodimas et al. 2004 [33]
Damos-08	$T=\mathrm{aa}\;\times \left(\frac{\mathrm{bb}-T}{10}\right)\times {\left(\frac{T}{10}\right)}^{\mathrm{cc}}$	Damos and Savopoulou-Soultani 2008 [17]

R_m_: maximum temperature; T: temperature; T_m_: optimum temperature; T_o_: rate at which temperature falls away from T_m_; a0, a1, a2, a3: constants; aa: constant; T_min_: minimum temperature; T_max_: maximum temperature; bb, cc: constants.

**Table 2:** Weighted mean developmental durations (days, mean ± SE) of *B. distincta* from egg to adult stage at different temperatures. Mean values with different letters indicate significant differences between temperatures in columns (*p* < 0.05). The number of individuals (N) that transitioned to the next stage is shown in brackets.

Developmental Stage								
Temperature, Relative Humidity	N	Egg	Instar 1	Instar 2	Instar 3	Instar 4	Instar 5	Egg to Adult
18 °C, 29%	241	15.0 ± 0.1 ^a^ (222)	14.2 ± 0.3 ^a^ (146)	-	-	-	-	-
21 °C, 91%	225	10.4 ± 0.1 ^b^ (192)	8.5 ± 0.1 ^b^ (158)	12.6 ± 0.3 ^a^ (101)	8.1 ± 0.3 ^a^ (83)	9.1 ± 0.4 ^ab^ (73)	11.8 ± 0.6 ^a^ (46)	60.3 ± 0.6 ^a^
22 °C, 30%	232	7.8 ± 0.1 ^c^ (232)	7.8 ± 0.1 ^b^ (199)	12.2 ± 0.3 ^a^ (99)	8.3 ± 0.5 ^a^ (90)	10.6 ± 0.4 ^a^ (79)	15.7 ± 0.6 ^b^ (60)	62.5 ± 0.6 ^a^
25 °C_high RH_, 64%	241	5.4 ± 0.1 ^d^ (232)	5.3 ± 0.1 ^c^ (214)	5.6 ± 0.1 ^b^ (158)	5.2 ± 0.1 ^b^ (128)	6.4 ± 0.2 ^bc^ (111)	10.4 ± 0.3 ^ac^ (82)	38.4 ± 0.3 ^b^
25 °C_low RH_, 25%	248	5.0 ± 0.0 ^d^ (243)	5.9 ± 0.1 ^c^ (204)	7.2 ± 0.1 ^ab^ (167)	5.3 ± 0.2 ^b^ (142)	7.4 ± 0.2 ^abc^ (121)	10.7 ± 0.2 ^ac^ (93)	41.6 ± 0.2 ^b^
29 °C, 23%	209	4.4 ± 0.1 ^d^ (194)	4.1 ± 0.1 ^c^ (157)	4.6 ± 0.1 ^b^ (98)	4.9 ± 0.1 ^b^ (94)	5.2 ± 0.2 ^c^ (90)	8.7 ± 0.2 ^c^ (77)	32.0 ± 0.4 ^c^
**Macadamia nut and sweetcorn diet comparison**								
25 °C, 71% (Macadamia nut)	194	6.2 ± 0.0 ^a^ (194)	5.2 ± 0.0 ^a^ (187)	7.2 ± 0.1 ^a^ (156)	6.3 ± 0.1 ^a^ (150)	7.0 ± 0.1 ^a^ (143)	10.6 ± 0.1 ^a^ (126)	43.3 ± 0.2 ^a^
25 °C, 64% (Sweetcorn)	241	5.4 ± 0.1 ^a^ (232)	5.3 ± 0.1 ^a^ (214)	5.6 ± 0.1 ^b^ (158)	5.2 ± 0.1 ^a^ (128)	6.4 ± 0.2 ^b^ (111)	10.4 ± 0.3 ^a^ (81)	38.5 ± 0.3 ^a^

**Table 3:** Survival rates (%, mean ± SE) of *B. distincta*’s life stages at different temperatures. Mean values with different letters indicate significant differences in survival between temperatures (*p* < 0.05).

Developmental Stage						
Temperature, Relative Humidity	Egg	Instar 1	Instar 2	Instar 3	Instar 4	Instar 5
18 °C, 29%	88 ± 0.0 ^a^	69 ± 0.1 ^a^	0.0 ± 0.0 ^a^	-	-	-
21 °C, 91%	95 ± 0.1 ^a^	76 ± 0.1 ^a^	66 ± 0.1 ^bc^	80 ± 0.1 ^a^	87 ± 0.0 ^a^	72 ± 0.1 ^a^
22 °C, 30%	100 ± 0.0 ^a^	86 ± 0.0 ^a^	51 ± 0.1 ^b^	88 ± 0.1 ^a^	88 ± 0.0 ^a^	76 ± 0.1 ^a^
25 °C_high RH_, 64%	96 ± 0.0 ^a^	92 ± 0.0 ^a^	73 ± 0.1 ^bc^	77 ± 0.1 ^a^	86 ± 0.0 ^a^	77 ± 0.1 ^a^
25 °C_low RH_, 25%	98 ± 0.0 ^a^	84 ± 0.1 ^a^	82 ± 0.1 ^c^	83 ± 0.0 ^a^	83 ± 0.0 ^a^	81 ± 0.1 ^a^
29 °C, 23%	93 ± 0.0 ^a^	81 ± 0.0 ^a^	64 ± 0.0 ^bc^	95 ± 0.0 ^a^	97 ± 0.0 ^a^	84 ± 0.1 ^a^
**Macadamia nut and sweetcorn diet comparison**						
25 °C, 71% (Macadamia nut)	95 ± 0.0 ^a^	96 ± 0.0 ^a^	83 ± 0.1 ^a^	96 ± 0.0 ^a^	95 ± 0.0 ^a^	88 ± 0.1 ^a^
25 °C, 64% (Sweetcorn)	96 ± 0.0 ^a^	92 ± 0.0 ^a^	73 ± 0.1 ^a^	77 ± 0.1 ^a^	86 ± 0.0 ^a^	77 ± 0.1 ^a^

**Table 4:** Estimated parameters (estimate ± SE) of the models fitted to the relationship between developmental rate and temperature of *B. distincta*’s life stages and statistical comparisons based on the coefficient of determination (R^2^) and Akaike Information Criterion (AIC). Models with the lowest AIC values are shown in bold for each life stage.

Developmental Stage							
Model	Parameters	Egg	Instar 1	Instar 2	Instar 3	Instar 4	Instar 5
Taylor-81	R_m_	0.23 ± 0.01 ***	0.23 ± 0.01 ***	0.21 ± 0.01 ***	0.21 ± 0.01 ***	0.17 ± 0.01 ***	0.11 ± 0.00 ***
	T_m_	27.9 ± 0.58 ***	28.4 ± 0.73 ***	28.4 ± 0.68 ***	27.1 ± 0.63 ***	28.0 ± 0.78 ***	27.5 ± 0.45 ***
	T_o_	−5.60 ± 0.48 ***	6.1 ± 0.59 ***	−4.84 ± 0.58 ***	5.24 ± 0.72 ***	6.13 ± 0.79 ***	6.36 ± 0.55 ***
	R^2^	0.04	0.005	0.006	0.02	0.007	0.03
	AIC	−143.1	−131.4	−114.8	**−105.8**	−118.5	−148.0
HarcourtYee-82	a0	0.83 ± 0.41.	9.96 ± 0.05 *	0.011 ± 0.046 *	0.011 ± 0.01	0.038 ± 0.04	0.0044 ± 0.025
	a1	−0.13 ± 0.04 **	−0.01 ± 0.00 **	−0.018 ± 0.00 **	−0.0047 ± 0.00	−0.0073 ± 0.00	−0.0021 ± 0.00
	a2	0.0073 ± 0.00 ***	0.00079 ± 0.00 ***	0.00088 ± 0.00 ***	0.00037 ± 0.00	0.00043 ± 0.00 *	0.00017 ± 0.00
	a3	−0.00011 ± 0.00 ***	−0.000013 ± 0.00 ***	−0.00001 ± 0.00 ***	−0.0000056 ± 0.00 *	−0.0000069 ± 0.00 **	−0.000031 ± 0.00 *
	R^2^	0.02	0.02	0.04	0.01	0.003	0.02
	AIC	−145.3	−137.3	−114.8	−103.7	−123.0	−152.5
Kontodimas-04	aa	0.00001 ± 0.00 ***	0.0000097 ± 0.00 ***	0.000012 ± 0.00 ***	0.0000084 ± 0.00 ***	0.0000069 ± 0.00 ***	0.0000036 ± 0.00 ***
	T_min_	0.011 ± 0.06 ***	0.013 ± 0.07 ***	0.015 ± 0.07 ***	0.011 ± 0.01 ***	0.012 ± 0.01 ***	0.011 ± 0.01 ***
	T_max_	0.038 ± 0.04 ***	0.038 ± 0.05 ***	0.038 ± 0.05 ***	0.037 ± 0.06 ***	0.038 ± 0.06 ***	0.038 ± 0.06 ***
	R^2^	0.02	0.02	0.04	0.005	0.003	0.02
	AIC	**−147.0**	−138.3	**−116.2**	−105.2	−125.0	**−154.2**
Damos-08	aa	0.003 ± 0.00 ***	0.0024 ± 0.00 ***	0.001 ± 0.00 *	0.0042 ± 0.00 *	0.0029 ± 0.00 **	0.002 ± 0.00 **
	bb	4.01 ± 0.03 ***	3.80 ± 0.03 ***	3.79 ± 0.03 ***	3.79 ± 0.06 ***	3.80 ± 0.04 ***	3.80 ± 0.05 ***
	cc	3.98 ± 0.15 ***	4.46 ± 0.27 ***	5.13 ± 0.40 ***	3.83 ± 0.40 ***	4.02 ± 0.33 ***	3.61 ± 0.31 ***
	R^2^	0.03	0.02	0.07	0.003	0.02	0.01
	AIC	−145.6	**−141.9**	−114.2	−102.8	**−125.9**	−153.9

Signif. codes: 0 *** 0.001 ** 0.01 * 0.05. 0.1. aa is the intercept of the line, bb: slope of the line, R_m_: maximum temperature rate, T_m:_ optimum temperature, T_o_: rate at which temperature falls away from T_m_. a0, a1, a2, a3: constant; aa, bb, cc: constant; T_min_: minimum temperature; T_max_: maximum temperature.

**Table 5:** Estimated developmental threshold parameters (lower: T_min_, optimum: T_opt_, and upper developmental threshold: T_max_) from the non-linear models using the “nlsDR” function from the DevRate package and thermal constant in degree days (DD) for each life stage of *B. distincta* estimated by the linear model.

Developmental Stage								
Model	Parameter (°C)	Egg	Instar 1	Instar 2	Instar 3	Instar 4	Instar 5	Egg to Adult
Taylor-81	T_min_	10.9	10.0	13.7	**11.2**	9.4	8.1	11.0
HarcourtYee-82		NE	NE	16.1	15.3	12.9	14.1	14.1
Kontodimas-04		**13.9**	13.6	**16.1**	12.8	12.9	**11.6**	**13.5**
Damos-08		7.9	**7.9**	9.6	6.2	**6.8**	5.6	7.2
Taylor-81	T_opt_	27.9	28.4	28.4	**27.1**	28.0	27.5	27.9
HarcourtYee-82		29.7	29.9	30.4	28.9	29.3	28.7	29.5
Kontodimas-04		**29.6**	29.9	**30.4**	29.2	29.3	**29.8**	**29.5**
Damos-08		30.9	**31.1**	31.8	30.1	**30.5**	29.8	30.6
Taylor-81	T_max_	NE	46.9	43.1	**43.1**	46.7	46.8	44.9
HarcourtYee-82		38.1	38.1	38.0	37.9	38.1	38.1	38.0
Kontodimas-04		**38.1**	38.2	**38.0**	37.9	38.1	**38.1**	**38.0**
Damos-08		38.0	**38.0**	38.0	37.9	**38.0**	38.0	38.0
Linear	DD	62.9	58.9	99.8	134.1	225.7	201.3	783.0

The values in bold represent the developmental thresholds estimated by the best models.

## Data Availability

The data supporting the findings of this study are available on request from the corresponding author.
